# Sample size determination for Bayesian ANOVAs with informative hypotheses

**DOI:** 10.3389/fpsyg.2022.947768

**Published:** 2022-11-22

**Authors:** Qianrao Fu, Mirjam Moerbeek, Herbert Hoijtink

**Affiliations:** ^1^School of Management, Xi'an University of Architecture and Technology, Xi'an, China; ^2^Department of Methodology and Statistics, Utrecht University, Utrecht, Netherlands

**Keywords:** Bayes factor, Bayesian ANOVAs, informative hypothesis, sample size, SSDbain

## Abstract

Researchers can express their expectations with respect to the group means in an ANOVA model through equality and order constrained hypotheses. This paper introduces the R package SSDbain, which can be used to calculate the sample size required to evaluate (informative) hypotheses using the Approximate Adjusted Fractional Bayes Factor (AAFBF) for one-way ANOVA models as implemented in the R package bain. The sample size is determined such that the probability that the Bayes factor is larger than a threshold value is at least η when either of the hypotheses under consideration is true. The Bayesian ANOVA, Bayesian Welch's ANOVA, and Bayesian robust ANOVA are available. Using the R package SSDbain and/or the tables provided in this paper, researchers in the social and behavioral sciences can easily plan the sample size if they intend to use a Bayesian ANOVA.

## 1. Introduction

In a classical one-way ANOVA, two hypotheses, the null hypothesis *H*_0_ and the alternative hypotheses *H*_*a*_ are contrasted:

(1) $$\begin{array}{l} H_{0}:\mu_{1}=\mu_{2}=\cdots =\mu_{K} \end{array}$$

vs.

(2) $$\begin{array}{l} H_{a}:\text{not all means are equal}, \end{array}$$

where μ_*k*_ denotes the mean for group *k* = 1, 2, …, *K*, and *K* denotes the number of groups.

Statistical power is the probability to correctly reject the null hypothesis when an effect exists in the population. Cohen (1988, 1992) published some of the most cited literature on power analysis; he proposed the effect size measure *f* = σ_*m*_/σ, where σ_*m*_ denotes the standard deviation of the means of the *K* groups, and σ the common within-group standard deviation. The classical sample size table of the one-way ANOVA based on the *F*-test (Cohen, 1992) indicates that in the case of three groups, 322, 52, or 21 subjects per group are needed to obtain a power of 0.8 to detect a small (*f* = 0.1), medium (*f* = 0.25), or large (*f* = 0.4) effect size at a Type I error rate α = 0.05. Required sample sizes for other scenarios can be calculated using software for power analysis and optimal study design, such as G*Power (Faul et al., 2007, 2009; Mayr et al., 2007), nQuery Advisor (Elashoff, 2007) and PASS (Hintze, 2011). Power analysis has become more important in a scientific world with competition for limited funding for research grants. Funding agencies often require value for money: if an effect size exists in the population then it should be detected with sufficient probability. However, many studies in the behavioral and social sciences are underpowered, mainly because of insufficient funding or numbers of subjects willing to participate. As well as a reduced probability of detecting an important effect size, underpowered research causes many problems, including overestimation of effect size, poor replicability of research findings, and thus an increased risk of drawing incorrect conclusions. For relevant articles, see Fraley and Vazire (2014), Maxwell (2004), Simonsohn et al. (2014), Dumas-Mallet et al. (2017), and Szucs and Ioannidis (2017).

Recently, null-hypothesis significance testing (NHST) has been criticized in numerous articles. Unnecessary detail will not be given in this paper, but see the typical references Nickerson (2000), Wagenmakers (2007), Masicampo and Lalande (2012), Harlow et al. (2016), and Wicherts et al. (2016). Alternatives such as Bayesian statistics have as a consequence become increasingly popular over the past decade (Van de Schoot et al., 2017; Vandekerckhove et al., 2018; Wagenmakers et al., 2016). Among them, Bayes factor is the most important tool to evaluate the competing hypotheses. The Bayes factor is the measurement of the relative evidence between two competing hypotheses. For example, if *H*_0_ vs. *H*_1_, and the Baye factor BF_01_ = 10, then the support for *H*_0_ is 10 times more than *H*_1_. The Bayes factor cannot only provide evidence in favor of the alternative hypothesis, but, in contrast to the *p*-value, also provides evidence in favor of the null hypotheses. The Bayes factor quantifies the strength of current data to support for *H*_0_ and *H*_1_, respectively, which is more balanced than the traditional NHST where Bayes factor are more balanced in terms of support for *H*_0_ and *H*_1_, and thus its tendency to reject *H*_0_ is relatively less strong. Under the traditional NHST hypothesis, as long as the collected data is enough the researcher can obtain *p* < 0.05 and thus reject *H*_0_, in contrast to the NHST, the Bayes factor tends to be stable with the increase of data. The Bayes factor does not depend on the unknown or nonexistent sampling plan, while the *p*-value is affected by the sampling plan. In addition, the traditional null and alternative hypotheses as specified by (1) and (2) may not reflect the researcher's expectations. The researcher can express his or her expectations with regard to the ordering of the group means μ_1_, μ_2_, …, μ_*K*_ in an informative hypothesis (Hoijtink, 2011). For example, consider a comparison of the average body heights of adults in the Netherlands, China, and Japan, as denoted by μ_*N*_, μ_*C*_ and μ_*J*_. Informative hypotheses may be formulated on the basis of observations, expectations or findings in the literature. One example is hypothesis *H*_1_ : μ_*N*_ > μ_*C*_ > μ_*J*_. It is worth mentioning that the Bayes factor can not only be used to compare the null hypothesis with alternative hypotheses, but also can be used to compare two informative hypotheses directly. Accordingly, in NHST if ordered hypothesis is included, multiple testing should be carried, which leads to increased chances of false positive results. Software for calculating Bayes factor are the R package BayesFactor, the R package BFpack, and the R package bain, which make the Bayes factor readily accessible to applied researchers. Therefore, it is important that sample size calculations for the Bayesian approach to hypothesis testing become available to researchers in the behavioral and social sciences.

Recently, a sequential Bayesian *t*-test (Schönbrodt et al., 2017) was developed that can, when applicable, avoid an a priori sample size calculation. A sequential test (Wald, 1945) allows researchers to add additional observations at every stage of an experiment depending on whether target strength of evidence is reached. That is, the size of the Bayes factor is large enough or a decision rule whether to i) accept the hypothesis being tested; ii) reject the hypothesis being tested; or iii) continue the experiment by making additional observations is satisfied.

However, a sequential test based on Bayesian updating is not always possible, for example, when the population of research is small (e.g., rare disease or cognitive disorder), when the study is longitudinal and runs for many years, when a research plan with an a priori sample size calculation is to be submitted to an ethical committee, or when researchers want to have an indication of the sample sizes needed even when they do use a sequential design. In these situations sample size determination is necessary. In practice, a combination of sample size determination and Bayesian updating is the best choice. For a more extensive overview of the role of sample size determination and Bayesian updating, the reader is referred to Fu et al. (2020).

Throughout this paper sample size determination (SSD) for the comparison of null, informative, and alternative hypotheses under a one-way ANOVA in the Bayesian framework van den Bergh et al. (2020), which will build on the sample size calculations for *t*-tests discussed in Schönbrodt and Wagenmakers (2018), Stefan et al. (2019), and Fu et al. (2020), will be performed. However, the observed data in social and behavioral research are often non-normal distributed or homogeneous of variance, see, for example, Glass et al. (1972), Micceri (1989), Harwell et al. (1992), Coombs et al. (1996), Keselman et al. (1998), and Blanca et al. (2013). To solve these problems, alternative ANOVAs will also be considered: (1) SSD for Bayesian Welch's ANOVA is available when homogeneity of variance does not hold; (2) SSD for Bayesian robust ANOVA is available when homogeneity of variance and normality of residuals do not hold and/or when the data contain outliers.

The outline of this paper is as follows. First, the models that are used in the article are introduced, the informative hypotheses that are evaluated is described, and the Approximate Adjusted Fractional Bayes Factor (AAFBF) approach as implemented in the R package bain is elaborated. Subsequently, sample size determination will be introduced, features of SSD will be highlighted, and examples will be provided and discussed. The paper ends with a short conclusion.

## 2. One-way ANOVAs, (Informative) hypotheses, and Bayes factor

In this paper, *K* mutually independent group means, μ_1_, μ_2_, ⋯ , μ_*K*_ are compared. Three different types of ANOVA models are considered:

Model 1: ANOVA, that is, the within-group variances for the *K* groups are equal

(3) $$\begin{array}{l} y_{tk}=\overset{K}{\underset{k=1}{\sum }}\mu_{k}D_{tk}+\epsilon_{tk},\epsilon_{tk}\sim N\left(0,\sigma^{2}\right), \end{array}$$

Model 2: Welch's ANOVA, that is, the within-group variances for the *K* groups are unequal

(4) $$\begin{array}{l} y_{tk}=\overset{K}{\underset{k=1}{\sum }}\mu_{k}D_{tk}+\epsilon_{tk},\epsilon_{tk}\sim N\left(0,\overset{K}{\underset{k=1}{\sum }}\sigma_{k}^{2}D_{tk}\right), \end{array}$$

Model 3: Robust ANOVA, that is, the within-group variances for the *K* groups are unequal, and the distribution of the residuals is non-normal and/or the data contain outliers

(5) $$\begin{array}{l} y_{tk}=\overset{K}{\underset{k=1}{\sum }}\mu_{k,ROB}D_{tk}+\epsilon_{tk},\epsilon_{tk}\sim f_{k}\left(\epsilon_{tk}\right), \end{array}$$

where *y*_*tk*_ for person *t* = 1, ⋯ , *N* belonging to group *k* = 1, 2, ⋯ , *K* is the dependent variable, *N* denotes the sample size per group, *D*_*tk*_ = 1 denotes that person *t* is a member of group *k* and 0 otherwise, ϵ_*tk*_ denotes the error in prediction for person *t* in group *k*, *f*_*k*_(ϵ_*tk*_) is an unspecified distribution of the residuals in group *k*, σ^2^ denotes the common within-group variance for each group in case of ANOVA, $\sigma_{k}^{2}$ denotes the within-group variance of group *k* in case of the Welch's ANOVA, and μ_*k,ROB*_ is the robust estimator of population mean.

In this paper, sample size will be determined under the following situations:

Situation 1: If the researchers believe that nothing is going on or something else is going on but they do not know what, sample size will be determined for the comparison of

*H*_0_ : μ_1_ = μ_2_ = ⋯ = μ_*K*_ vs. *H*_*a*_, where *H*_*a*_: not all means are equal;

Situation 2: Many researchers have clear ideas or expectations with respect to what might be going on. These researchers might believe nothing is going on or have a specific expectation about the ordering of the means. Therefore sample size will be determined for a comparison of

*H*_0_ : μ_1_ = μ_2_ = ⋯ = μ_*K*_ vs. $H_{i}:\mu_{1^{*}}>\mu_{2^{*}}>\cdots >\mu_{K^{*}}$;

where 1^*^, 2^*^, ⋯ , K^*^ are a re-ordering of the numbers 1, 2, ⋯ , *K*;

Situation 3: Or, continuing Situation 2, researchers may want to compare their expectation with its complement. Therefore sample size will be determined for a comparison of

$H_{i}:\mu_{1^{*}}>\mu_{2^{*}}>\cdots >\mu_{K^{*}}$ vs. *H*_*c*_: not *H*_*i*_;

Situation 4: The researchers have two competing expectations

$H_{i}:\mu_{1^{*}}>\mu_{2^{*}}>\cdots >\mu_{K^{*}}$ vs. $H_{j}:\mu_{1^{\#}}>\mu_{2^{\#}}>\cdots >\mu_{K^{\#}}$,

where 1^#^, 2^#^, ⋯ , *K*^#^ denote a re-ordering of numbers 1, 2, ⋯ , *K* that is different from *H*_*i*_. Note that, SSD is also possible if some of the ">" in *H*_*i*_ or *H*_*j*_ are replaced by "=."

The AAFBF as implemented in the R package bain will be used to determine the relative support in the data for a pair of hypotheses. The interested reader is referred to Gu et al. (2018), Hoijtink et al. (2019a) and Hoijtink et al. (2019b) for the complete statistical background. Here only the main features of this approach will be presented. If, for example, BF_*ij*_ = 10, this implies that the data are 10 times more likely to have been observed under *H*_*i*_ than under *H*_*j*_. In this manuscript, the AAFBF will be used because it is currently the only Bayes factor available that can handle the four situations introduced above for regular ANOVA, Welch's ANOVA, and robust ANOVA. In what follows, the AAFBF implementation for ANOVAs will be described. First of all, the Bayes factor with which *H*_0_ and *H*_*i*_ can be compared to *H*_*a*_ will be introduced. Subsequently, BF_*ij*_ and BF_*ic*_ will be introduced.

Let *H*_*z*_ denote either of *H*_0_ and *H*_*i*_, and note that for robust ANOVA **μ** has to be replaced by **μ**_*ROB*_, then

(6) $$\begin{array}{l} {\text{BF}}_{za}=\frac{f_{z}}{c_{z}}=\frac{\int_{\mu \in H_{z}}g_{a}\left(\mu \right)d\mu }{\int_{\mu \in H_{z}}h_{a}\left(\mu \right)d\mu } \end{array}$$

where *f*_*z*_ and *c*_*z*_ are the fit and complexity of *H*_*z*_ relative to *H*_*a*_, respectively, *g*_*a*_(**μ**) denotes a normal approximation to the posterior distribution of **μ** under *H*_*a*_, and *h*_*a*_(**μ**) the corresponding prior distribution of **μ** under *H*_*a*_. The fit is the proportion of the posterior distribution *g*_*a*_(·) in agreement with *H*_*z*_, and the complexity is the proportion of the prior distribution *h*_*a*_(·) in agreement with *H*_*z*_. The Bayes factor (BF) for *H*_*i*_ against *H*_*j*_ is:

(7) $${\text{BF}}_{ij}=\frac{{\text{BF}}_{ia}}{{\text{BF}}_{ja}}=\frac{f_{i}/c_{i}}{f_{j}/c_{j}},$$

and the BF of *H*_*i*_ vs. *H*_*c*_ is:

(8) $${\text{BF}}_{ic}=\frac{{\text{BF}}_{ia}}{{\text{BF}}_{ca}}=\frac{f_{i}/c_{i}}{\left(1-f_{i}\right)/\left(1-c_{i}\right)}.$$

The posterior distribution used in the AAFBF is a normal approximation of the actual posterior distribution of the *K* group means. This can be justified using large sample theory (Gelman et al., 2013, pp. 101). This normal approximation can be specified using the estimates of μ, the residual variance *s*^2^ and *N*. For the regular ANOVA (Model 1) this renders:

(9) $$\begin{array}{l} g_{a}\left(\mu \right)=\iint_{\mu \in \mu }\pi_{a}\left(\mu ,\sigma^{2}\right)d\mu \;d\sigma^{2}\\ =\int_{\mu \in \mu }\pi_{a}\left(\mu \right)d\mu =N\left(\left[\begin{array}{c} \hat{\mu } \end{array}\right],\left[\begin{array}{cc} {ŝ}^{2}/N & 0\\ \ddots \\ 0 & {ŝ}^{2}/N \end{array}\right]\right); \end{array}$$

for the Welch's ANOVA (Model 2) this renders:

(10) $$\begin{array}{l} g_{a}\left(\mu \right)=N\left(\left[\begin{array}{c} \hat{\mu } \end{array}\right],\left[\begin{array}{cc} {ŝ}_{1}^{2}/N & 0\\ \;\ddots \\ 0 & {ŝ}_{K}^{2}/N \end{array}\right]\right); \end{array}$$

where $\hat{\mu }=\left[{\hat{\mu }}_{1},{\hat{\mu }}_{2},\cdots \;,{\hat{\mu }}_{K}\right]$ denotes the maximum likelihood estimates of the *K* group means, ŝ^2^ denotes the unbiased estimate of the residual variance, and ${ŝ}_{1}^{2}$, ${ŝ}_{2}^{2}$, ⋯ , ${ŝ}_{K}^{2}$ denote unbiased estimates of the *K* within-group variances. For the robust ANOVA (Model 3),

(11) $$\begin{array}{l} g_{a}\left(\mu \right)=N\left(\left[\begin{array}{c} {\hat{\mu }}_{\text{ROB}} \end{array}\right],\left[\begin{array}{cc} {ŝ}_{1,ROB}^{2}/N & 0\\ \;\ddots \\ 0 & {ŝ}_{K,ROB}^{2}/N \end{array}\right]\right). \end{array}$$

where ${\hat{\mu }}_{ROB}$ is the 20% trimmed mean, which according to Wilcox (2017, pp. 45-93) is the best choice, and ${ŝ}_{k,ROB}^{2}$ is a robust estimate of the residual variance in Group *k*, which is based on the Winsorized variance (see, Wilcox, 2017, pp. 60–64). If the data are severely non-normal or contain outliers, the estimates of means can be very poor estimates of central tendency, and the within-group variances can be very poor estimates of the variability within a group (Bosman, 2018) therefore in these situations it may be preferable to use ${\hat{\mu }}_{ROB}$ and ${ŝ}_{k,ROB}^{2}$ for *k* = 1, ⋯ , *K*.

The prior distribution is based on the adjusted (Mulder, 2014) fractional Bayes factor approach (O'Hagan, 1995). As is elaborated in Gu et al. (2018) and Hoijtink et al. (2019a) for the regular ANOVA with homogeneous within-group variances (Model 1), the prior distribution is:

(12) $$\begin{array}{l} h_{a}\left(\mu \right)=N\left(\left[\begin{array}{c} 0 \end{array}\right],\left[\begin{array}{cc} \frac{1}{b}\times \frac{{ŝ}^{2}}{N} & 0\\ \;\ddots \\ 0 & \frac{1}{b}\times \frac{{ŝ}^{2}}{N} \end{array}\right]\right); \end{array}$$

and, for the Welch's ANOVA with group specific variances (Model 2) the prior distribution is

(13) $$\begin{array}{l} h_{a}\left(\mu \right)=N\left(\left[\begin{array}{c} 0 \end{array}\right],\left[\begin{array}{cc} \frac{1}{b}\times \frac{{ŝ}_{1}^{2}}{N} & 0\\ \;\ddots \\ 0 & \frac{1}{b}\times \frac{{ŝ}_{K}^{2}}{N} \end{array}\right]\right); \end{array}$$

and, for the robust ANOVA (Model 3) the prior distribution is

(14) $$\begin{array}{l} h_{a}\left(\mu \right)=N\left(\left[\begin{array}{c} 0 \end{array}\right],\left[\begin{array}{cc} \frac{1}{b}\times \frac{{ŝ}_{1,ROB}^{2}}{N} & 0\\ \;\ddots \\ 0 & \frac{1}{b}\times \frac{{ŝ}_{K,ROB}^{2}}{N} \end{array}\right]\right). \end{array}$$

For the hypotheses considered in this paper mean of the prior distribution should be the origin **0**. As is elaborated in Mulder (2014), this choice renders a quantification of complexity in accordance with Occam's razor and, as is elaborated in Hoijtink et al. (2019b), it renders a Bayes factor that is consistent. The variances appearing in the prior distribution are based on a fraction of the information in the data. For each group in an ANOVA this fraction is $b=\frac{J}{K}\times \frac{1}{N}$ (Hoijtink et al., 2019a). The choice for the parameter *J* is inspired by the minimal training sample approach (Berger and Pericchi, 1996, 2004): it is the number of independent constraints used to specify the hypotheses under consideration, because these can be seen as the number of underlying parameters (the differences between pairs of means) that are of interest. Specifically, if *H*_0_ : μ_1_ = μ_2_ = μ_3_ vs *H*_*i*_ : μ_1_ > μ_2_ > μ_3_ is considered, *J* is equal to 2. The choice for minimum training samples is to some degree arbitrary. It is in general common in Bayesian analyzes to execute sensitivity (to the prior distribution) analyzes. Hence alternative choices of $b=\frac{2J}{K}\times \frac{1}{N}$ and $b=\frac{3J}{K}\times \frac{1}{N}$ are also considered in this paper. Note that, prior sensitivity only applies to Situations 1 and 2, the Bayes factors computed for Situations 3 and 4 are not sensitive to the choice of *b* (see Mulder, 2014).

## 3. Sample size determination for one-way ANOVAs

SSD for the Bayesian one-way ANOVA is implemented in the R package SSDbain^1^. This section describes the specific ingredients needed for the functions SSDANOVA and SSDANOVA_robust in the R package SSDbain. The interested reader is referred to Appendices A,B for an elaboration of the SSD algorithm. After installing the R package SSDbain, the following Call 1 and Call 2 are used to calculate the sample size per group for regular ANOVA and Welch's ANOVA:

Call 1: using Cohen's *f* (Cohen, 1992) to specify the populations of interest


**#load SSDbain package**
**library(SSDbain)**
**SSDANOVA(hyp1="mu1=mu2=mu3",hyp2="Ha", type="equal",f1**
**     =0,f2=0.25,var=NULL,**
**BFthresh=3,eta=0.8,T=10000,seed=10)**


Call 2: using means and variances to specify the populations of interest


**#load SSDbain package**
**library(SSDbain)**
**SSDANOVA(hyp1="mu1=mu2=mu3",hyp2="Ha",type="equal",f1=**
     **c(0,0,0),f2=**
**c(5.5,4.5,2),var=c(4,4,4),BFthresh=3,eta=0.8,T=10000,**
     **seed=10)**


and the Call 3 below is used for a robust ANOVA:


**#load SSDbain package**
**library(SSDbain)**
**SSDANOVA_robust(hyp1="mu1=mu2=mu3",hyp2="Ha",f1=0,f2**
     **=0.25,skews=c(0,0,0),**
**kurts=c(0,0,0),var=c(1.5,0.75,0.75),BFthresh=3,eta**
     **=0.8,T=10000,seed=10)**


The following arguments appear in these calls:

1. hyp1 and hyp2, strings that specify the hypotheses of interest. If the unconstrained hypothesis is used, hyp2="Ha;" if the complement hypothesis is used, hyp2="Hc." In case of three groups the default setting is hyp1="mu1=mu2=mu3," and hyp2="mu1>mu2>mu3," which generalizes seamlessly to more than three groups.
2. type, a string that specifies the type of ANOVA. If one expects that the *K* within-group variances are equal, type="equal," otherwise type="unequal."
3. f1 and f2, parameters used to specify the populations corresponding to hyp1 and hyp2, respectively. There are two options. In Call 1 given above f1 and f2 denote Cohen's *f* = σ_μ_/σ where σ_μ_ denotes the standard deviation of the means of the *K* groups, and σ denotes the common within-group standard deviation. If type = "equal," the var=NULL is required, where var = NULL denotes that the variances do not have to be specified. If type = "unequal," the var has to be specified by the users (see the next argument for detail). In Call 2 given above f1 and f2 contain the population means corresponding to both hypotheses hyp1 and hyp2. This option can always be used and requires the specification of var. In Call 3, the combination of Cohen's *f* and within-group variances or the combination of means and variances are used to specify the populations of interest. In Appendix C it is elaborated how population means are computed if f1 and f2 denote Cohen's *f*.
4. var, vector of length *K* that specifies the within-group variances of the *K* groups. If type = "equal" and *f*_1_ and *f*_2_ are Cohen's *f*, the specification var = NULL implies that each within-group variance is set to 1. In case of type = "unequal" or Call 3, the user needs to input Cohen's *f* and the variances for each group. The corresponding population means can be computed. In Appendix C it is elaborated how in both cases the corresponding population means are computed.
5. skews and kurts, vectors of length *K* that specify the skewness and kurtosis for the *K* groups compared. Here kurtosis means the true kurtosis minus 3, that is, the kurtosis is 0 when the distribution is normal. The default setting is skews=c(0,0,0) and kurts=c(0,0,0), which renders a normal distribution. Note that the relationship kurtosis ≥ skewness^2^ − 2 should hold (Shohat, 1929).
  Two situations can be distinguished. If researchers want to execute an ANOVA that is robust against outliers, both skews and kurts are zero vectors with dimension *K*. Outliers can be addressed in this manner because robust estimates of the mean and its variance obtained for data sampled from a normal distribution (that is, without outliers) are very similar to the robust estimates obtained for data sample from a normal distribution to which outliers are added. If researchers want to address skewed or heavy tailed data, they have to specify the expected skewness and kurtosis for each group.
  The following gives guidelines for choosing appropriate values for skewness and kurtosis. If the population distribution is left-skewed, the skewness is a negative value; if the population distribution is right-skewed, the skewness is a positive value. The commonly used example of a distribution with a positive skewness is the distribution of salary data where many employees earn relatively little, while just a few employees have a high salary. In addition, typical response time data often show positive skewness because long response times are less common (Palmer et al., 2011). The high school GPA of students who apply for college often shows a negative skewness. Furthermore, in psychological research, scores on easy cognitive tasks tend to be negatively skewed because the majority of participants can complete most tasks successfully (Wang et al., 2008). If the population distribution is heavy-tailed relative to a normal distribution, the kurtosis is larger than 0; if the population distribution has lighter tailed than a normal distribution, the kurtosis is smaller than 0.
  The values to be used for the skewness and kurtosis can be chosen based on a meta-analysis or literature review (e.g., Schmidt and Hunter, 2015). The absolute value of the skewness is typically smaller than 3 in psychological studies. As a general rule, skewness and kurtosis values that are within ±1 of the normal distribution's skewness of 0 and kurtosis of 0 indicate sufficient normality. Blanca et al. (2013) studied the shape of the distribution used in the real psychology, and found that 20% of the distribution showed extreme non-normality. Therefore, it is essential to consider robust ANOVA when the non-normal distribution is involved. After determining the values of the skewness and kurtosis relevant for their populations, researchers can use SSDANOVA_robust to determine the sample sizes needed for a robust evaluation of their hypotheses for data sampled from populations that skewed and/or show kurtosis. The non-normal data is generated from a generalization of the normal distribution that accounts for skewness and kurtosis. The Tukey *g*-and-*h* family of non-normal distributions (see, Headrick et al., 2008; Jorge and Boris, 1984) is commonly used for univariate real data generation in Monte Carlo studies. If the researchers input the skewness and kurtosis, *g* and *h* can be obtained (Headrick et al., 2008). The data can be generated as follows. Firstly, *T* (see point 8 for a explanation on Page 18) data sets with sample size *N* from the standard distribution are simulated; secondly, observations are transformed into a sample from the *g*-and-*h*-distribution as below
  if *g* ≠ 0
  (15) $$\begin{array}{l} T\left(X\right)=A+B\exp\left(h/2X^{2}\right)\left(\exp\left(gX\right)-1\right)/g \end{array}$$
  if *g* = 0
  (16) $$\begin{array}{l} T\left(X\right)=A+B\exp\left(h/2X^{2}\right)X \end{array}$$
  where *X* ~ *N*(0, 1), *A* is the mean parameter, *B* is the standard deviation parameter, *g* is the skewness parameter, and *h* is the kurtosis parameter.

### 3.1. Intermezo: The probability that the Bayes factor is larger than a threshold value

**Figure 1:**
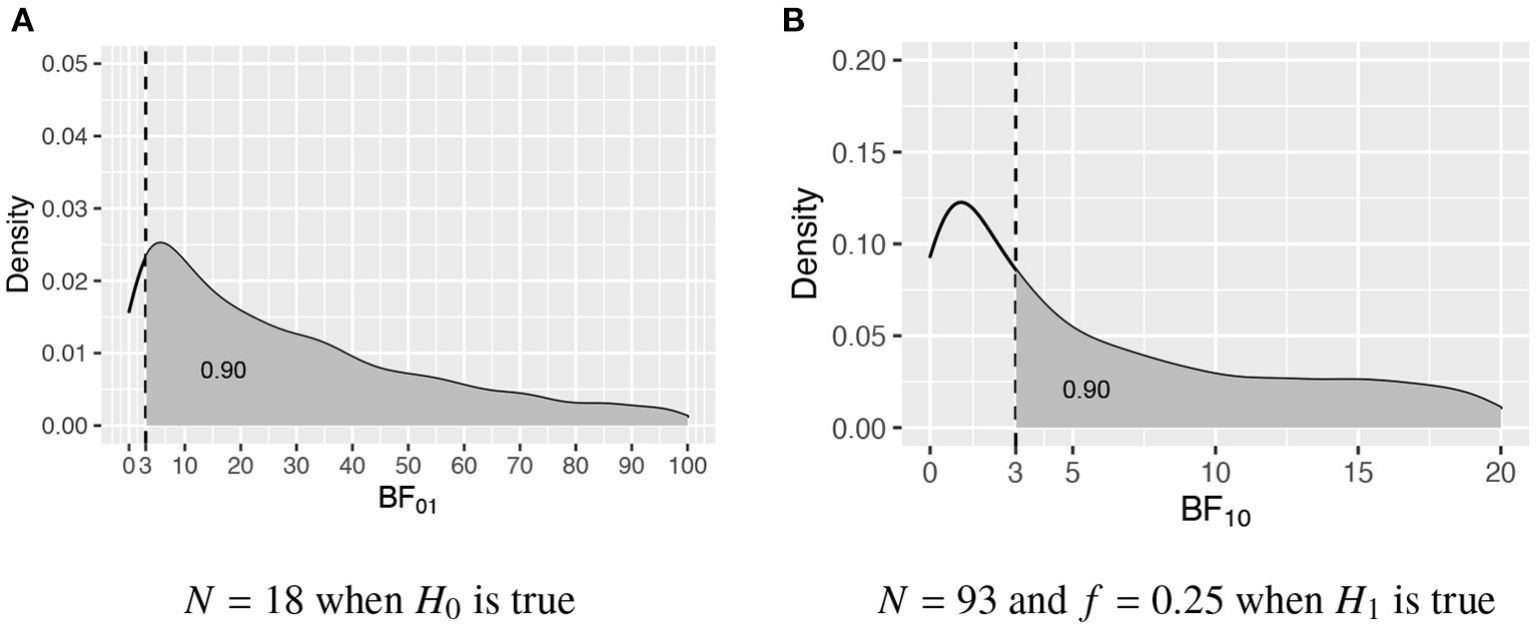
The sampling distribution of BF_01_ under *H*_0_ and BF_10_ under *H*_1_. The vertical dashed line represents BF_*thresh*_ = 3, and the gray area denotes η, that is, the probability that the Bayes factor is larger than 3. **(A)** *N* = 18 when *H*_0_ is true. **(B)** *N* = 93 and *f* = 0.25 when *H*_1_ is true.

In this intermezzo it will be elaborated how the required sample size is determined once the populations corresponding to the two competing hypotheses have been specified, that is, once the population group means, variances, and possibly skewness and kurtosis have been specified. Figure 1 portrays the distributions of the Bayes factor under *H*_0_ : μ_1_ = μ_2_ = μ_3_ and *H*_1_ : μ_1_ > μ_2_ > μ_3_, that is, when data are repeatedly sampled from *H*_0_ and for each data set BF_01_ is computed, what is the distribution of BF_01_, and, when data are repeatedly sampled from *H*_1_ and for each data set BF_10_ is computed, what is the distribution of BF_10_. Figure 1A shows the distribution obtained using *N* = 18 per group, and Figure 1B shows the distribution obtained using *N* = 93 per group. To determine these sample sizes, two criteria are specified. First of all, what is the required size of the Bayes factor to be denoted by BF_*thresh*_; and, secondly, what should be the minimum probability that BF_01_ and BF_10_ are larger than BF_*thresh*_ denoted by *P*(BF_01_ > BF_*thresh*_|*H*_0_) ≥ η and *P*(BF_10_ > BF_*thresh*_|*H*_1_) ≥ η, respectively. As can be seen in Figure 1, BF_*thresh*_ = 3 and η = 0.90, that is, with *N* = 18 *P*(BF_01_ > 3|*H*_0_) ≥ 0.90, and with *N* = 93 *P*(BF_10_ > 3|*H*_1_) ≥ 0.90. Therefore, to fulfill the criteria for both *H*_0_ and *H*_1_, *N* = 93 persons per group are required.

Two aspects of sample size determination need to be elaborated: how to choose BF_*thresh*_ and how to choose η. The choice of the BF_*thresh*_ is subjective, common values are 3, 5, and 10. In high-stakes research, such as a clinical trial to compare a new medication for cancer to a placebo and a standard medication, one would prefer a large BF_*thresh*_. In low-stakes research, such as an observational study on the comparison of ages of customers at three different coffeehouses, one may use a smaller BF_*thresh*_. The second is how to determine η. It should be noted that 1-η is the Bayesian counterpart of the Type I error rate if hyp1 is true, and the Bayesian counterpart of the Type II error rate if hyp2 is true. If the consequences of failing to detect the effect could be serious, such as in toxicity testing, one might want a relatively high η such as 0.90. In studies where one may only be interested in large effects, an error for detecting the effect may not have such serious consequences. Here an η = 0.80 may be sufficient.

6. BFthresh, a numeric value not less than 1 that specifies the required size of the Bayes factor. The default setting is BFthresh=3.
7. eta, a numeric value that specifies the probability that the Bayes factor is larger than BFthresh if either of the competing hypotheses is true. The default setting is eta=0.80.
8. T, a positive integer that specifies the number of data sets sampled from the populations corresponding to the two hypotheses of interest. A larger number of samples returns a more precise sample size estimate but takes longer to run. We recommend that users start with a smaller number of samples (e.g., T = 1,000) to get a rough estimate of sample size before confirming it with the default setting T = 10,000.
9. seed, a positive integer that specifies the seed of R's random number generator. It should be noted that different data sets are simulated in Step 8 if a different seed is used, and thus, that the results of sample size determination may be slightly different. However, the sample sizes obtained using two different seeds give an indication of the stability of the results (this will be highlighted when discussing Table 4 in Appendix). The default setting is seed=10.

The results of the functions SSDANOVA and SSDANOVA_robust include the sample size required per group and the corresponding probability that the Bayes factor is larger than BF_*thresh*_ when either of the competing hypotheses is true. For example, if the following call to SSDANOVA is executed


**library(SSDbain)**
**SSDANOVA(hyp1 = "mu1=mu2=mu3, "hyp2 = "Ha," type = "**
     **equal," f1 = 0, f2 = 0.25, var = NULL,**
**BFthresh = 3, eta=0.8, T = 10000, seed = 10)**


the results for *b* based on the minimum value of *J*, and the results for *b* based on 2*J* and 3*J* (with the aim to address the sensitivity to the specification of the prior distribution) are:


**     using *****N***** = 93 and *****b***** = 0.007**
**     P(BF0a>3|H0)=0.977**
**     P(BFa0>3|Ha)=0.801**
**     using *****N***** = 83 and *****b***** = 0.016**
**     P(BF0a>3|H0)=0.949**
**     P(BFa0>3|Ha)=0.802**
**     using *****N***** = 77 and *****b***** = 0.026**
**     P(BF0a>3|H0)=0.918**
**     P(BFa0>3|Ha)=0.802**


Further interpretation of the results of SSD will be given in the form of three examples that will be presented after the next section.

## 4. Features of sample size determination for one-way ANOVAs

In this section sample sizes are given based on classical hypotheses, informative hypotheses, and their complement hypotheses for one-way ANOVAs with three groups when the effect size is Cohen's *f* = 0.1, *f* = 0.25, and *f* = 0.4. Table 1 in Appendix shows the populations corresponding to *H*_1_, *H*_2_, *H*_*a*_, and *H*_*c*_ for three different effect sizes when the pooled within-group variance is 1. Tables 2–5 in Appendix show the sample size and the corresponding probability that the Bayes factor is larger than BF_*thresh*_ for regular, Welch's and robust ANOVA for *H*_0_ vs. *H*_*a*_, *H*_0_ vs. *H*_1_, *H*_1_ vs. *H*_2_, and *H*_1_ vs. *H*_*c*_, respectively. Table 6 in Appendix displays the robust ANOVA for moderately skewed, extremely skewed, and heavy tailed populations. All the tables are obtained with set.seed=10. To illustrate the stability of the results when using T = 10,000, in Table 4 in Appendix additionally the results are obtained using set.seed=1234. Based on the results presented in these tables a number of features of SSD will be highlighted.

Comparing Table 3 in Appendix with Table 2 in Appendix, it can be seen that the sample size required is smaller if *H*_0_ is compared to the order constrained hypothesis *H*_1_ instead of to the unconstrained hypothesis *H*_*a*_. For example, if effect size *f* = 0.25, BF_*thresh*_ = 3, η = 0.8, and regular ANOVA are chosen, the sample size required is 93 per group if *H*_0_ is compared to *H*_*a*_, while the sample size required is 71 per group if *H*_0_ is compared to *H*_1_. This is because *H*_1_ is more precise than *H*_*a*_ and it is easier to find evidence against or for a more precise hypothesis.

Comparing Table 4 in Appendix with Table 3 in Appendix, it can be clearly seen that the comparison of two non-nested hypotheses like *H*_1_ and *H*_2_ requires less sample size than the comparison of nested hypotheses like *H*_0_ and *H*_1_ (*H*_0_ is in fact on the boundary of *H*_1_). For example, if effect size *f* = 0.25, BF_*thresh*_ = 3, η = 0.8, and regular ANOVA is used, the sample size required is 71 per group if *H*_0_ is compared to *H*_1_, while the sample size required is 13 per group for *H*_1_ is compared to *H*_2_. The same phenomenon can be observed comparing Table 4 in Appendix (*H*_1_ vs. *H*_2_) with Table 5 in Appendix (*H*_1_ vs. *H*_*c*_). Although in both cases non-nested hypotheses are compared, *H*_2_ is much more precise than *H*_*c*_ and therefore the required sample size for the comparison of *H*_1_ with *H*_2_ is smaller than for the comparison of *H*_1_ with *H*_*c*_. In summary the more specific the hypotheses that are evaluated, the smaller the required sample size. The sample size is further reduced if two non-nested hypotheses are compared.

From Tables 2–5 in Appendix, it appears that the sample size required is smaller for a regular ANOVA than for a Welch's ANOVA. For example, as shown in Table 2 in Appendix, if effect size *f* = 0.25, BF_*thresh*_ = 3, η = 0.8, and *H*_0_ vs. *H*_*a*_, the sample size required for regular ANOVA is 93 per group, while the sample size required is 102 per group for Welch's ANOVA. However, this is not always the case. The sample size required for Welch's ANOVA may be smaller than the sample size required for a regular ANOVA. The main determinant is order of the size of the variances relative to the order of the means.

For the robust ANOVA, two situations are evaluated. First of all, if the data may include outliers, Tables 2–5 in Appendix apply, because sampling from a normal distribution and using 20% trimming is a very good approximation of sampling from a normal with outliers. Secondly, if the data is skewed or heavy tailed, the results in Table 6 in Appendix apply. Three situations are distinguished: skewness = 0.61 and kurtosis = 0.67, skewness = 1.75 and kurtosis = 5.89, and skewness = 0 and kurtosis = 6.94. These three situations represent moderately skewed, extremely skewed, and extremely heavy-tailed distributions that are often encountered in psychological research (Micceri, 1989; Cain et al., 2017). From Tables 2–5 in Appendix, it can be seen that the sample size required is the largest for robust ANOVA. Comparing Table 3 in Appendix in which the data had a skewness of 0 and a kurtosis of 0 with Table 6 in Appendix, it can be seen that the required sample sizes are larger if robust ANOVA is used to evaluate hypotheses using data sampled from skewed and heavy tailed population distributions.

In addition, the extremely skewed distribution needs smaller sample size than moderately skewed, and the extremely heavy tailed needs a higher sample size than skewed.

Finally, as is illustrated in Table 4 in Appendix, when T = 1,0000 is used, the results of SSD are very stable, that is, the required sample sizes and η_1_ and η_2_ are irrelevantly different if different seeds are used. This was also observed for the other tables but these results are not reported in this paper.

## 5. Examples of sample size determination for one-way ANOVAs

To demonstrate how to use the functions SSDANOVA and SSDANOVA_robust to execute SSD for one-way ANOVAs in practice, in the following we introduce three practical examples. The first example presents the SSD process for the regular ANOVA, the second example presents the SSD process for the Welch's ANOVA, and the third example presents the SSD process for the robust ANOVA.

Example 1: A team of researchers in the field of educational science wants to conduct a study in the area of mathematics education involving different teaching methods to improve standardized math scores. The study will randomly assign fourth grade students who are randomly sampled from a large urban school district to three different teaching methods. The teaching methods are 1) The traditional teaching method where the classroom teacher explains the concepts and assigns homework problems from the textbook; 2) the intensive practice method, in which students fill out additional work sheets both before and after school; 3) the peer assistance learning method, which pairs each fourth grader with a fifth grader who helps them learn the concepts. At the end of the semester all students take the Multiple Math Proficiency Inventory (MMPI). The researchers expect that the traditional teaching group (Group 1) will have the lowest mean score and that the peer assistance group (Group 3) will have the highest mean score. That is,

*H*_1_ : μ_3_ > μ_2_ > μ_1_.

This hypothesis is compared to *H*_0_ which states that the standardized math scores are the same in the three conditions.

*H*_0_: μ_1_ = μ_2_ = μ_3_.

The researchers guess a priori that Group 1 has a mean of 550, Group 2 has a mean of 560, and Group 3 has a mean that equals 580. Based on prior research, the common standard deviation σ is set to 50. Therefore the effect size is $f=\frac{\sigma_{m}}{\sigma }=0.249$. The researchers decide to use BF_*thresh*_ = 3 because they are happy to get some evidence in favor of the best hypothesis. They also choose η = 0.8 because their research is not a high-stakes research. The researchers also want to do a sensitivity analysis to see how the sample size is influenced by *b*. To determine the required sample size the researchers use the following call to SSDANOVA.


**library(SSDbain)**
**SSDANOVA(hyp1="mu1=mu2=mu3," hyp2 = "mu3>mu2>mu1,"**
**     type = ''equal,'' f1 = (0,0,0),**
**f2=c(550,560,580), var = c(2,500,2,500,2,500),**
**     BFthresh=3,eta=0.8, T = 10000,**
**seed=10)**


The results are as follows:


**using N = 73 and b = 0.009**
**P(BF03>3|H0)=0.972**
**P(BF30>3|H3)=0.801**
**using N = 62 and b = 0.021**
**P(BF03>3|H0)=0.944**
**P(BF30>3|H3)=0.803**
**using N = 55 and b = 0.036**
**P(BF03>3|H0)=0.909**
**P(BF30>3|H3)=0.802**


According to the results the researchers should execute their project using between 55 and 73 persons per group. These are the numbers that they can submit to the (medical) ethical review committee, and, to which they should tailor their resources (time, effort and money). The researchers can combine the results of SSD with Bayesian updating (see the elaboration on this topic in Fu et al., 2020) to avoid using too few or too many persons. Bayesian updating can be executed as follows. They can use 1/4 of the sample size 73, that is, collect 18 students per group firstly, and compute the Bayes factor once the data have been collected. If the Bayes factor is larger than 3, they stop the experiment; otherwise, they collect another 18 students per group, compute the Bayes factor using 36 students per group, and check if the Bayes factor is larger than 3, etc. In this manner, resources can be used in an optimal way while reaching the required amount of evidence.

Example 2: A team of psychologists is interested in whether male college students' hair color (1: black, 2: blond, or 3: brunette) influences their social extroversion. The students are given a measure of social extroversion with a range from 0 (low) to 10 (high). Based on a meta analysis of research projects addressing the same research question, the means in the three groups are specified as 7.33, 6.13, and 5.00, and the standard deviations are 2.330, 2.875, and 2.059, respectively. The sampling variance which is denoted as 'var' in the following code is the squared of standard deviation. The effect size is $f=\frac{\sigma_{m}}{\sigma }=0.39$. The researchers want to replicate the result emerging of the existing body of evidence, that is, is it *H*_1_: μ_1_ > μ_2_ > μ_3_ or *H*_*c*_: not *H*_1_. They want to obtain decisive evidence BF_*thresh*_ = 10 with a high probability η = 0.90. The researchers use the following call to SSDANOVA:


**library(SSDbain)**
**SSDANOVA(hyp1="mu1>mu2>mu3," hyp2="Hc," type=''unequal**
**     ,'' f1=c(7.33,6.13,5.00),**
**f2=c(5.00,7.33,6.13), var=c(2.330^2,2.875^2,2.059^2),**
**     BFthresh=10, eta=0.9,**
**T = 10000, seed=10)**


The results are as follows:


**using N = 38 and b = 0.017**
**P(BF1c>3|H1)=0.903**
**P(BFc1>3|Hc)=0.988**


Therefore the researchers should obtain 38 males for each hair color.

Example 3: A team of economists would like to conduct a study to compare the average salary of three age groups in the US. The typical salary distribution in an age group population usually shows positive skewness. Three age groups that include 25-34, 35-44, and 45-54 years old are considered, and the mean salaries for these three groups are denoted as μ_1_, μ_2_, and μ_3_, respectively. Based on prior research, experts' opinion or a pilot study, they assume the effect size is *f* = 0.25, the variances are 1.5, 0.75, and 0.75, the skewnesses are 2, 2.5, and 1.75, and the kurtosis is 6, 10, and 6, respectively. The researchers are only interested in a decision for or against one of the two hypotheses involved. Therefore they use BF_*thresh*_ = 1 and use η = 0.90 to have a high probability that the observed Bayes factor correctly identifies the best hypothesis. Two hypotheses are involved: *H*_1_:μ_2_ > μ_3_ > μ_1_ and *H*_2_:μ_3_ > μ_2_ > μ_1_. The following call is used:


**library(SSDbain)**
**SSDANOVA_robust(hyp1="mu2>mu3>mu1," hyp2="mu3>mu2>mu1,**
**     " f1=0.25,f2=0.25,skews=**
**c(2,2.5,1.75),kurts=c(6,10,6),var=c(1.5,0.75,0.75),**
**     BFthresh=1,eta=0.9,**
**T = 10000, seed=10)**



**using N = 50 and b = 0.013**
**P(BF23>1|H2)=0.976**
**P(BF32>1|H3)=0.904**


The results show that if the researchers survey 50 persons per group, they have a probability that the Bayes factor is larger than 1 of 0.976 if *H*_1_ is true or get a probability that the Bayes factor is larger than 1 of 0.904 if *H*_2_ is true.

## 6. Conclusion

In this paper we introduced sample size determination for the evaluation of the classical null and alternative hypotheses and informative hypotheses (and their complement) in the one way ANOVA context, using the AAFBF as is implemented in the R package bain. Our SSD approach is implemented in the functions SSDANOVA (which covers regular ANOVA and Welch's ANOVA) and SSDANOVA_robust (which covers robust ANOVA) which are part of the R package SSDbain. Besides the one-way ANOVA, SSDbain also contains the function SSDttest (Fu et al., 2020). In the near future another function, SSDregression, will be added to evaluate (informative) hypotheses using the Bayes factor in the context of multiple regression models. We believe that the R package SSDbain is a welcome addition to the applied researcher's toolbox, and may help the researcher to get an idea about the required sample sizes while planning a research project. The novelty of this research can be concluded as follows:

1. A new sample size determination principle is proposed. Different from traditional unilateral principle, we give a principle, which can be described as the probability that the Bayes factor is larger than a threshold value is at least? when either of the hypotheses under consideration is true.
2. A sample size determination method based on dichotomy is proposed, which can effectively reduce the computation effort. In the traditional sample size determination method, the sample size is increase by 1 until the termination conditions are satisfied. This method is simple and easy to be implemented. However, it might be very time-consuming especially when the sample size is very large. The dichotomy-based sample size determination method only requires a small number of iterations, which is more convenient to the practical application.
3. The sample size determination method proposed in this paper has wider applicability. The software developed in this paper is available for Bayesian ANOVA, Bayesian Welch's ANOVA, and Bayesian robust ANOVA.

The usage of informative hypothesis results in a reduction in the number of sample size required, which further saves the resources. However, Given the sample size requirement for informative hypotheses is usually lower, the researchers may choose to plan their studies with an informative hypothesis even when there is no strong evidence for the specified direction of the means, just so that they can justify their small sample size. This may further exacerbate the replicability crisis problems in the literature. Therefore, the user should be careful if the informative hypothesis is introduced.

## Data availability statement

The original contributions presented in the study are included in the article/Supplementary material, further inquiries can be directed to the corresponding author/s.

## Author contributions

QF, MM, and HH designed the research. QF developed the software package and wrote the paper. MM and HH gave feedback on software development, constructing, and writing the paper. All authors contributed to the article and approved the submitted version.

## Funding

QF was supported by the China Scholarship Council. HH was supported by a fellowship from the Netherlands Institute for Advanced Study in the Humanities and Social Sciences (NIAS-KNAW) and the Consortium on Individual Development (CID) which was funded through the Gravitation program of the Dutch Ministry of Education, Culture, and Science and the Netherlands Organization for Scientific Research (NWO Grant No. 024.001.003).

## Conflict of interest

The authors declare that the research was conducted in the absence of any commercial or financial relationships that could be construed as a potential conflict of interest.

## Publisher's note

All claims expressed in this article are solely those of the authors and do not necessarily represent those of their affiliated organizations, or those of the publisher, the editors and the reviewers. Any product that may be evaluated in this article, or claim that may be made by its manufacturer, is not guaranteed or endorsed by the publisher.

## Footnotes

^1^SSDbain comes with a user manual and can be installed from https://github.com/Qianrao-Fu/SSDbain. Further information on bain can be found at https://informative-hypotheses.sites.uu.nl/software/bain/.
