# Supplementary material for: Sample size determination for Bayesian ANOVAs with informative hypotheses
Source: Front Psychol. 2022 Nov 22;13:947768. doi: 10.3389/fpsyg.2022.947768 (PMC9724823; doi:10.3389/fpsyg.2022.947768)
Supplement: Supplementary file 1 [file Data_Sheet_1.pdf]

Appendix A: Basic Algorithm used in Bayesian SSD for one-way ANOVAs

The workflow of the sample size determination is presented in Figure 2. The basic algorithm used to determine the sample size uses the following steps:

1. Researchers have to specify the nine ingredients discussed in the section "Sample Size Determination for One-Way ANOVAs".
2. Simulate T data sets with sample size $N = 10$ per group from each of the two populations defined by the specifications given under 1. The data sets are denoted as $D_s^1, D_s^2, \cdots, D_s^T$, and $D_v^1, D_v^2, \cdots, D_v^T$, where s can be represented as 0 or i, and v can be represented as a, j or c.
3. Compute the Bayes factor (regular ANOVA, Welch's ANOVA, or robust ANOVA) for each simulated data set. If H_s is true the Bayes factor is denoted by $\mathtt{BF}_{sv}^t$, if H_v is true, the Bayes factor is denoted by $\mathtt{BF}_{vs}^t$. Subsequently the probability $P(\mathrm{BF}_{sv} > \mathrm{BF}_{thresh}|H_s)$ denoted as η_s and the probability $P(\mathrm{BF}_{vs} > \mathrm{BF}_{thresh}|H_v)$ denoted as η_v can be computed.
4. If both η_s and η_v are larger than η, the algorithm stops and the results are provided. Otherwise, the sample size N is increased by 1 and the algorithm restarts in Step 2.

To execute a sensitivity analyses Steps 1 through 4 are not only executed using fraction $b = \frac{J}{K}\frac{1}{N}$ but also using $b = \frac{2J}{K}\frac{1}{N}$ and $b = \frac{3J}{K}\frac{1}{N}$. SSD may take a large amount of time. In order to calculate the sample size efficiently, an improved algorithm based on a dichotomy algorithm is introduced below.

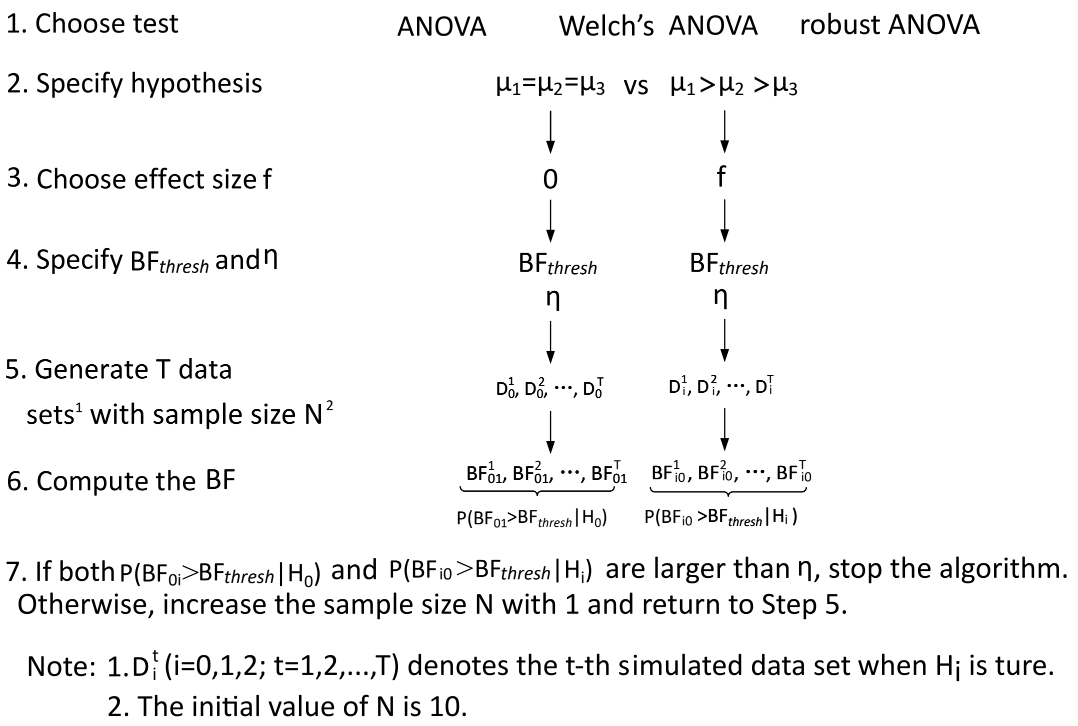

Figure 1. The workflow of the sample size determination.

Appendix B: An Improvement of the Basic Algorithm

In this appendix the refinement that makes the basic algorithm faster is described. It is computer intensive to iterate Steps 2-4 many times until the conditions in Step 4 are satisfied. The number of iterations will be reduced and the calculation time will be shorter if Step 2-4 from the basic algorithm are replaced by the steps presented below. The basic principle of Steps 6-8 is to gradually adjust the sample size using a dichotomy algorithm until $P(\mathrm{BF}_{sv} > \mathrm{BF}_{thresh}|H_s) \geq \eta$ and $P(\mathrm{BF}_{vs} > \mathrm{BF}_{thresh}|H_v) \geq \eta$ hold. Figure 3 portrays a flowchart to help the reader have a visual representation of the sequence of steps:

2. Set the initial sample size $N = 100$.

3. Generate $t = 1, \cdots, T$ data sets with sample size N per group from each of the two populations, respectively. The data sets are denoted as $D_s^1, D_s^2, \cdots, D_s^T$, and $D_v^1, D_v^2, \cdots, D_v^T$.

4. Calculate the corresponding T BFs under the T data sets, respectively, denoted as BF_{sv}^t

$(t = 1, 2, \ldots, T)$, and BF_{vs}^{t}. Then the probability $P(\mathrm{BF}_{sv} > \mathrm{BF}_{thresh}|H_s)$ denoted as η_s and the probability $P(\mathrm{BF}_{vs} > \mathrm{BF}_{thresh}|H_v)$ denoted as η_v can be computed.

5. If both η_s and η_v are larger than η, set $N = \frac{N}{2}$. Return to Step 3 and repeat until one or both of η_s and η_v are smaller than η. At this time, let $N_{\mathrm{min}} = N$, $N_{\mathrm{max}} = 2 * N$. If one or both of η_s and η_v are smaller than η, set $N = 2 * N$. Return to Step 3 and repeat until both η_s and η_v are larger than η. At this time, let $N_{\mathrm{min}} = \frac{N}{2}$, $N_{\mathrm{max}} = N$.
6. Set $N = N_{\mathrm{mid}} = (N_{\mathrm{min}} + N_{\mathrm{max}})/2$, and perform Steps 3-4.
7. If both η_s and η_v are larger than η, set $N_{\mathrm{max}} = N_{\mathrm{mid}}$; Otherwise, set $N_{\mathrm{min}} = N_{\mathrm{mid}}$.
8. Repeat Step 6 until $N_{\mathrm{mid}} = N_{\mathrm{min}} + 1$. The final sample size is N_{mid}.

Appendix C: Practical Motivations of Informative Hypotheses

Informative hypotheses are formulated based on the assumptions and expectations of the researcher or the findings and conclusions in the literature. The practical motivations of informative hypotheses in the experimental designs when calculating the sample size can be concluded as follows:

1. The specific expectations and questions of a researcher can be expressed by informative hypotheses. For instance, when the means for different populations, groups, conditions or treatments are compared, the regression coefficients are compared and the sign of the regression coefficient is judged. For example, researchers want to study the effects of tea on weight loss, and form three groups: green tea, black tea, and herbal tea, with the mean weight loss in these groups denoted by μ_{green}, μ_{black} and μ_{herbal}, respectively. They obtain the expectation about the ordering of the effects of these three types of teas from previous studies. This expectation can be expressed as H_1: $\mu_{\mathrm{green}} > \mu_{\mathrm{black}} > \mu_{\mathrm{herbal}}$.
2. Evaluation of informative hypotheses can eliminate the multiple testing problem that occurs when one needs follow-up tests to unravel an omnibus effect in null hypothesis significance

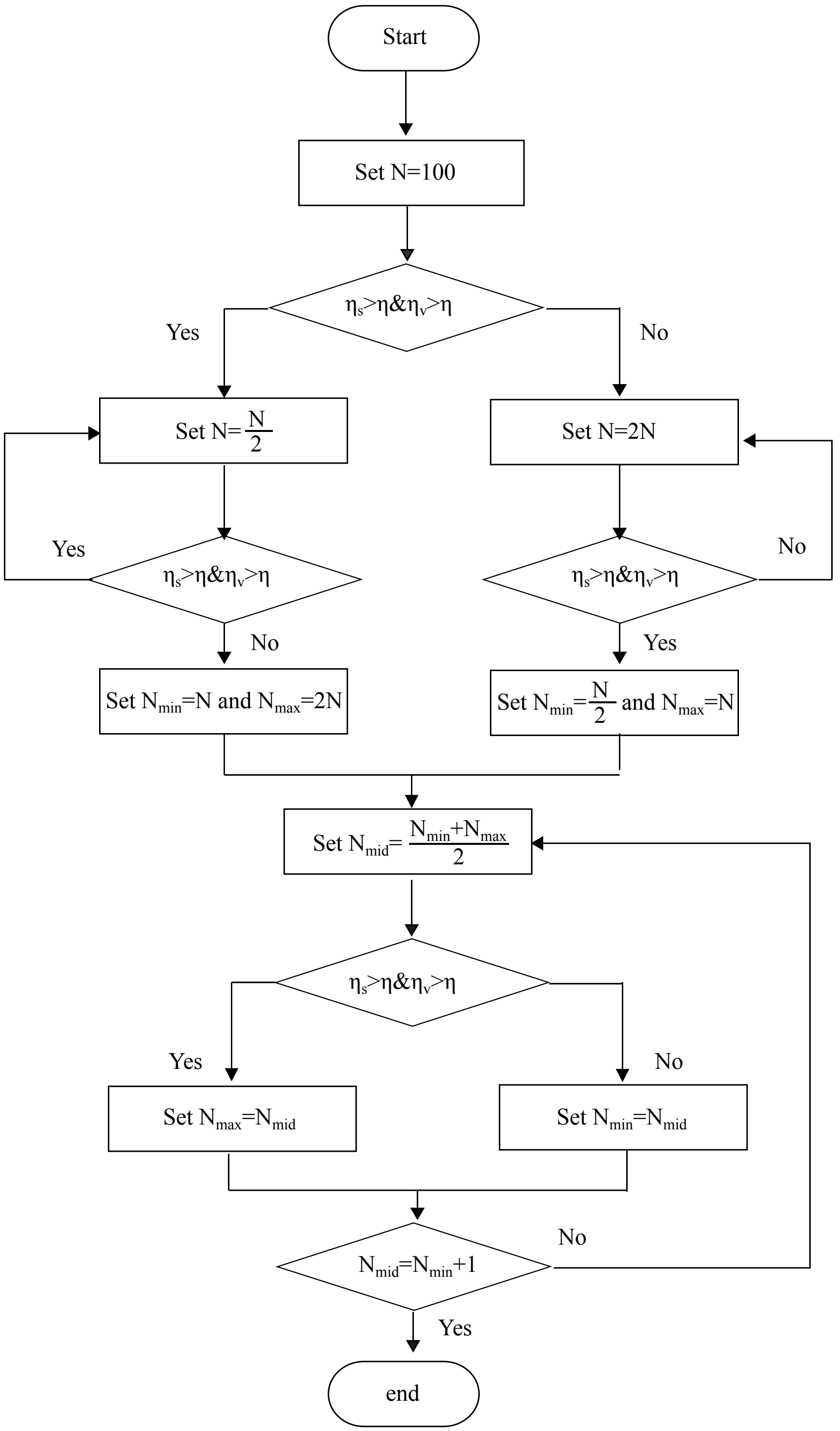

Figure 2. An improvement of the basic algorithm: Sample size determination for the Bayesian one-way ANOVA. Note that $\eta_s = P(\mathrm{BF}_{sv} > \mathrm{BF}_{thresh}|H_s)$, $\eta_v = P(\mathrm{BF}_{vs} > \mathrm{BF}_{thresh}|H_v)$.

testing. For example, an increased Type I error rate and the loss of power that results from adjustments for multiple testing (**maxwell2004persistence**) can be avoided. To continue the previous example, testing H_0 versus H_a: not H_0, requires follow-up tests in the form of pairwise comparisons of means if H_0 is rejected in favor of H_1: $\mu_{\text{green}}>\mu_{\text{black}}>\mu_{\text{herbal}}$, the follow-up tests are not needed. While making the effort to specify informative hypotheses, researchers will study the literature, think, and engage in academic debate. This will force them to carefully consider the hypotheses and what can and cannot be concluded when hypotheses are (not) supported. This should result in better hypotheses and, after their evaluation, in better additions to the theory in the research field of interest.

3. Using an informative hypothesis can result in a smaller sample size than using an unconstrained hypothesis. To illustrate this, the required sample size for the null hypothesis H_1: $\mu_{\text{green}}=\mu_{\text{black}}=\mu_{\text{herbal}}$ versus an alternative hypothesis H_a is calculated with the `R` package `SSDbain`, which is 93, while it is 71 for the null hypothesis H_0: $\mu_{\text{green}}=\mu_{\text{black}}=\mu_{\text{herbal}}$ versus an inequality hypothesis H_1: $\mu_{\text{green}}>\mu_{\text{black}}>\mu_{\text{herbal}}$ when the effect size of Cohen's f=0.25 is used. The sample sizes in the table are computed using BF_{thresh}=3 and η=0.8. It can be concluded that the required sample size is reduced if H_0 is not compared to H_a but to an informative hypothesis H_1.

Appendix D: The Impact of Effect Size

In this paper, the effect size is set as 0.1, 0.25, and 0.4 for the small, medium and large effect size. Actually, this effect size is only a rough estimate based on previous experience or literature information. Therefore, the sensitivity analysis of the violations of the effect size should be discussed for the calculation of sample size and the probability that the Bayes factor is larger than a threshold value. We still take the effects of tea on weight loss as example. The sample size is 71 for the null hypothesis H_0: $\mu_{\text{green}}=\mu_{\text{black}}=\mu_{\text{herbal}}$ versus an inequality hypothesis H_1: $\mu_{\text{green}}=\mu_{\text{black}}=\mu_{\text{herbal}}$, when the effect size of Cohen's f=0.25 is used. However, the actual effect size often deviates from the estimated value. Figure 4a presents the influence of the violations of

the effect size on the calculated sample size when $\text{BF}_{thresh} = 3$ and h=0.8. Figure 4b shows the influence of the violations of the effect size on the calculation of the probability that the Bayes factor is larger than a threshold value $P(\text{BF}_{01} > \text{BF}_{thresh} \& \text{BF}_{10} > \text{BF}_{thresh})$ when sample size is 71. It can be seen that with the increase of the effect size, there will be a decrease for the sample size, and an increase for the probability. However, a sample size that can be used as a reference for the researcher is given with the sample size determination method proposed in this paper. For example, if the effect size is assumed to be 0.25, and its actual value is 0.24, we will get a smaller sample size than the required value. Then, we can use the Bayesian updating to get the actual sample size conveniently.

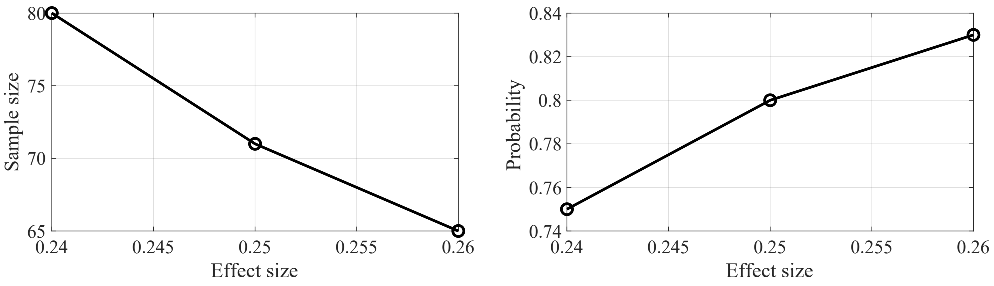

(a) Sample size (b) Probability

Figure 3. Sample size and probability with the variation of effect size.

Appendix D: How to determine the means based on an effect size

In the functions `SSDANOVA` and `SSDANOVA_robust` of the `R` package `SSDbain`, if the researchers specify a Cohen's effect size f, for regular ANOVA it is assumed that the within-group variance $\sigma^2 = 1$, and for Welch's ANOVA and robust ANOVA, the within-group variance σ^2 is set equal to the average of the within-groups variances the user entered for each of the groups. Then the means are determined automatically based on the given effect size f and the within-group variance.

In the following we will introduce how to determine the means for K groups if H_0, H_a, H_i, or H_c is true.

For the null hypothesis H_0, the effect size is $f = 0$, and the default population mean for each group

is zero.

For the unconstrained hypothesis H_a, the default population means are in order $\mu_1 > \mu_2 > \cdots > \mu_K$. If, for example, $K = 4$, we assume $(\mu_1, \mu_2, \mu_3, \mu_4)$=$(3d, 2d, d, 0)$. Based on the formula $f = \sigma_\mu/\sigma = \sqrt{\frac{1}{4}\sum_1^4(\mu_i - \bar{\mu})^2}/\sigma = \sqrt{\frac{1}{4} * 5d^2}/\sigma$, the value of d can be obtained, and thus the population means can be computed.

For the order hypothesis H_i: $\mu_{1^*} > \mu_{2^*} > ... > \mu_{K^*}$, the default population means are in order $\mu_{1^*} > \mu_{2^*} > ... > \mu_{K^*}$. If, for example, $H_i : \mu_1 > \mu_3 > \mu_2 > \mu_4$, we assume $(\mu_1, \mu_2, \mu_3, \mu_4)$ is equal to $(3d, d, 2d, 0)$. Based on the formula $f = \sigma_\mu/\sigma = \sqrt{\frac{1}{4}\sum_1^4(\mu_i - \bar{\mu})^2}/\sigma = \sqrt{\frac{1}{4} * 5d^2}/\sigma$, the value of d can be computed and thus the population means can be computed.

If the hypothesis is H_i, the complemented hypotheses can be divided into $\binom{K}{2}$ categories based on the adjacent pairs of violation of the means, where $\binom{K}{2}$ is a combinatorial number. For ease of understanding, two simple examples for $K = 3$ and $K = 4$ are given:

Example 1: H_1: $\mu_1 > \mu_2 > \mu_3$ vs H_c

(1 pair of violation): H_{c1}: $\mu_2 > \mu_1 > \mu_3$, $H_{c2} : \mu_1 > \mu_3 > \mu_2$;

(2 pairs of violations): H_{c3}: $\mu_3 > \mu_1 > \mu_2$, H_{c4}: $\mu_2 > \mu_3 > \mu_1$;

(3 pairs of violations): H_{c5}: $\mu_3 > \mu_2 > \mu_1$.

The Bayes factor BF_{c1} for H_c vs H_1 becomes larger with the increase of the number of pairs of violation for the complemented population from H_1. Furthermore, the Bayes factor BF_{c1} under population H_{c3} is smaller than under population H_{c4}. The median number hypothesis H_{c3} of H_{ci} $(i = 1, \cdots, 5)$ is chosen as the representative hypothesis to simulate data under H_c, that is, the means of the complement hypothesis are in the order $\mu_3 > \mu_1 > \mu_2$. For this hypothesis the means can be computed as was done earlier for H_i.

Example 2: H_1:$\mu_1 > \mu_2 > \mu_3 > \mu_4$ vs H_c

(1 pair of violation): H_{c1}: $\mu_2 > \mu_1 > \mu_3 > \mu_4$, H_{c2}: $\mu_1 > \mu_3 > \mu_2 > \mu_4$, H_{c3}:

$\mu_1 > \mu_2 > \mu_4 > \mu_3$;

(2 pairs of violations): H_{c4}: $\mu_2 > \mu_3 > \mu_1 > \mu_4$, H_{c5}: $\mu_2 > \mu_1 > \mu_4 > \mu_3$, H_{c6}: $\mu_1 > \mu_3 > \mu_4 > \mu_2$, H_{c7}: $\mu_3 > \mu_1 > \mu_2 > \mu_4$; H_{c8}: $\mu_1 > \mu_4 > \mu_2 > \mu_3$;

(3 pairs of violations): H_{c9}: $\mu_3 > \mu_2 > \mu_1 > \mu_4$, H_{c10}: $\mu_2 > \mu_3 > \mu_4 > \mu_1$, H_{c11}: $\mu_2 > \mu_4 > \mu_1 > \mu_3$, H_{c12}: $\mu_3 > \mu_1 > \mu_4 > \mu_2$, H_{c13}: $\mu_1 > \mu_4 > \mu_3 > \mu_2$, H_{c14}: $\mu_4 > \mu_1 > \mu_2 > \mu_3$;

(4 pairs of violations): H_{c15}: $\mu_3 > \mu_2 > \mu_4 > \mu_1$, H_{c16}: $\mu_2 > \mu_4 > \mu_3 > \mu_1$, H_{c17}: $\mu_4 > \mu_2 > \mu_1 > \mu_3$, H_{c18}: $\mu_3 > \mu_4 > \mu_1 > \mu_2$, H_{c19}: $\mu_4 > \mu_1 > \mu_3 > \mu_2$;

(5 pairs of violations): H_{c20}: $\mu_3 > \mu_4 > \mu_2 > \mu_1$, H_{c21}: $\mu_4 > \mu_2 > \mu_3 > \mu_1$, H_{c22}: $\mu_4 > \mu_3 > \mu_1 > \mu_2$;

(6 pairs of violations): H_{c23}: $\mu_4 > \mu_3 > \mu_2 > \mu_1$

As described in the previous example, the Bayes factor BF_{c1} for H_c vs H_1 becomes larger with the increase of pairs of violation for the complemented population from H_1. Furthermore, the Bayes factors BF_{c1} under population H_{ci} ($i = 9, \cdots, 14$) are sorted in ascending order. The median number hypothesis H_{c12} of H_{ci} ($i = 1, \cdots, 23$) is chosen as the representative hypothesis to simulate data under H_c, that is, the means of the complement hypothesis are in the order $\mu_3 > \mu_1 > \mu_4 > \mu_2$. For this hypothesis the means can be computed as was done earlier for H_i.

Table 1
The populations that are used to determine sample size

situations	f = 0.1					f = 0.25					f = 0.4				
	μ_1	μ_2	μ_3	γ	κ	μ_1	μ_2	μ_3	γ	κ	μ_1	μ_2	μ_3	γ	κ
H_1: $\mu_1 > \mu_2 > \mu_3$	0.2450	0.1225	0.0000	0	0	0.6124	0.3062	0.0000	0	0	0.9798	0.4899	0.0000	0	0
	0.2450	0.1225	0.0000	0.61	0.67	0.6124	0.3062	0.0000	0.61	0.67	0.9798	0.4899	0.0000	0.61	0.67
	0.2450	0.1225	0.0000	1.75	5.89	0.6124	0.3062	0.0000	1.75	5.89	0.9798	0.4899	0.0000	1.75	5.89
	0.2450	0.1225	0.0000	0	6.94	0.6124	0.3062	0.0000	0	6.94	0.9798	0.4899	0.0000	0	6.94
H_2: $\mu_2 > \mu_3 > \mu_1$	0.0000	0.2450	0.1225	0	0	0.0000	0.6124	0.3062	0	0	0.0000	0.9798	0.4899	0	0
H_a: μ_1, μ_2, μ_3	0.2450	0.1225	0.0000	0	0	0.6124	0.3062	0.0000	0	0	0.9798	0.4899	0.0000	0	0
H_c: not H_1	0.0000	0.2450	0.1225	0	0	0.0000	0.6124	0.3062	0	0	0.0000	0.9798	0.4899	0	0

Note: For hypothesis $H_0 : \mu_1 = \mu_2 = \mu_3$, the means are (0, 0, 0) for the three populations. For regular ANOVA, the σ^2 equals 1, for Welch's ANOVA and for robust ANOVA, σ_k^2 for $k = 1, 2, 3$ equals 1.5, 0.75 and 0.75, respectively. The highlight rows denote the populations used in Table 6, and the others denote the populations used in Tables 2-5. Note that skewness is denoted as γ, kurtosis is denoted as κ, and Cohen's f equals $\frac{\sigma_\mu}{\sigma}$, where σ denotes the pooled within-group standard deviation.

Table 2
For hypotheses $H_0 : \mu_1 = \mu_2 = \mu_3$ vs $H_a : \mu_1, \mu_2, \mu_3$, the required sample size N per group, and the corresponding $\eta_0 = P(\mathrm{BF}_{0a} > 3|H_0)$ and $\eta_a = P(\mathrm{BF}_{a0} > 3|H_a)$.

effect size			$f = 0.1$				$f = 0.25$				$f = 0.4$			
η			0.80		0.90		0.80		0.90		0.80		0.90	
fraction	type of ANOVA	hypotheses	N	η_0/η_a	N	η_0/η_a	N	η_0/η_a	N	η_0/η_a	N	η_0/η_a	N	η_0/η_a
$b = \frac{1}{K}\frac{J}{N}$	equal	H_0	756	0.997	924	0.999	93	0.977	119	0.982	31	0.926	41	0.947
		H_a		0.800		0.901		0.801		0.905		0.807		0.910
	unequal	H_0	822	0.998	1004	0.998	102	0.979	127	0.982	34	0.934	44	0.946
		H_a		0.801		0.902		0.802		0.901		0.809		0.910
	robust	H_0	965	0.998	1170	0.999	120	0.981	150	0.985	40	0.931	55	0.954
		H_a		0.800		0.900		0.813		0.902		0.815		0.921
$b = \frac{1}{K}\frac{2J}{N}$	equal	H_0	692	0.995	861	0.996	83	0.949	107	0.960	27	0.843	44	0.901
		H_a		0.800		0.901		0.802		0.905		0.812		0.954
	unequal	H_0	750	0.995	924	0.998	90	0.950	115	0.963	29	0.850	46	0.903
		H_a		0.801		0.902		0.802		0.903		0.809		0.949
	robust	H_0	879	0.996	1080	0.996	105	0.958	135	0.966	35	0.861	46	0.900
		H_a		0.801		0.902		0.803		0.907		0.825		0.905
$b = \frac{1}{K}\frac{3J}{N}$	equal	H_0	655	0.991	821	0.992	77	0.918	99	0.932	32	0.811	62	0.900
		H_a		0.802		0.900		0.802		0.900		0.897		0.995
	unequal	H_0	706	0.992	884	0.994	83	0.923	107	0.939	32	0.805	65	0.903
		H_a		0.802		0.902		0.805		0.903		0.874		0.993
	robust	H_0	825	0.993	1038	0.994	100	0.931	125	0.945	32	0.809	58	0.903
		H_a		0.800		0.900		0.817		0.901		0.803		0.962

Table 3

For hypotheses $H_0 : \mu_1 = \mu_2 = \mu_3$ vs $H_1 : \mu_1 > \mu_2 > \mu_3$, the required sample size N per group, and the corresponding $\eta_0 = P(\mathrm{BF}_{01} > 3|H_0)$ and $\eta_1 = P(\mathrm{BF}_{10} > 3|H_1)$.

effect size			$f = 0.1$				$f = 0.25$				$f = 0.4$			
η			0.80		0.90		0.80		0.90		0.80		0.90	
fraction	type of ANOVA	hypotheses	N	η_0/η_1	N	η_0/η_1	N	η_0/η_1	N	η_0/η_1	N	η_0/η_1	N	η_0/η_1
$b = \frac{1}{K}\frac{J}{N}$	equal	H_0	611	0.996	761	0.998	71	0.971	93	0.980	22	0.922	31	0.939
		H_1		0.802		0.901		0.805		0.901		0.803		0.908
	unequal	H_0	664	0.997	830	0.997	78	0.976	101	0.980	24	0.924	33	0.941
		H_1		0.802		0.900		0.806		0.900		0.802		0.902
	robust	H_0	785	0.998	975	0.998	91	0.978	120	0.984	30	0.925	40	0.947
		H_1		0.802		0.900		0.806		0.908		0.828		0.911
$b = \frac{1}{K}\frac{2J}{N}$	equal	H_0	546	0.992	694	0.995	60	0.943	81	0.956	17	0.820	35	0.901
		H_1		0.801		0.900		0.807		0.901		0.800		0.964
	unequal	H_0	598	0.993	751	0.995	66	0.942	89	0.956	19	0.828	38	0.902
		H_1		0.801		0.901		0.807		0.903		0.806		0.963
	robust	H_0	700	0.996	885	0.997	80	0.953	105	0.964	25	0.852	37	0.905
		H_1		0.802		0.900		0.819		0.906		0.836		0.920
$b = \frac{1}{K}\frac{3J}{N}$	equal	H_0	514	0.989	655	0.991	52	0.901	75	0.927	23	0.804	52	0.901
		H_1		0.802		0.902		0.800		0.904		0.910		0.997
	unequal	H_0	559	0.990	706	0.994	58	0.910	81	0.935	24	0.805	54	0.901
		H_1		0.801		0.900		0.802		0.903		0.897		0.996
	robust	H_0	655	0.992	840	0.993	70	0.915	96	0.941	23	0.812	53	0.907
		H_1		0.802		0.901		0.813		0.902		0.815		0.985

Table 4

For hypotheses $H_1 : \mu_1 > \mu_2 > \mu_3$ vs $H_2 : \mu_2 > \mu_3 > \mu_1$, the required sample size N per group, and the corresponding $\eta_1 = P(\mathrm{BF}_{12} > 3|H_1)$ and $\eta_2 = P(\mathrm{BF}_{21} > 3|H_2)$.

effect size		$f = 0.1$				$f = 0.25$				$f = 0.4$			
η		0.80		0.90		0.80		0.90		0.80		0.90	
type	hypotheses	N	η_1/η_2	N	η_1/η_2	N	η_1/η_2	N	η_1/η_2	N	η_1/η_2	N	η_1/η_2
equal	H_1	80 (80)	0.805 (0.801)	139 (141)	0.901 (0.901)	13 (13)	0.808 (0.810)	22 (22)	0.904 (0.901)	10 (10)	0.921 (0.925)	10 (10)	0.921 (0.925)
	H_2		0.800 (0.808)		0.902 (0.904)		0.806 (0.808)		0.902 (0.900)		0.923 (0.929)		0.923 (0.929)
unequal	H_1	103 (103)	0.811 (0.802)	173 (176)	0.905 (0.900)	16 (17)	0.808 (0.810)	28 (28)	0.907 (0.904)	10 (10)	0.888 (0.891)	11 (11)	0.902 (0.904)
	H_2		0.803 (0.806)		0.900 (0.901)		0.804 (0.812)		0.903 (0.909)		0.884 (0.891)		0.903 (0.904)
robust	H_1	114 (117)	0.804 (0.800)	200 (203)	0.905 (0.906)	20 (20)	0.824 (0.824)	33 (31)	0.906 (0.904)	10 (10)	0.871 (0.871)	13 (14)	0.902 (0.910)
	H_2		0.801 (0.807)		0.901 (0.902)		0.822 (0.821)		0.906 (0.904)		0.866 (0.874)		0.900 (0.910)

Note: in this table, the fraction $b = \frac{1}{K}\frac{J}{N}$ is used because the results are independent of the choice of b (Mulder, 2014). The numbers outside the brackets are based on `set.seed=10`, the numbers in the brackets are based on `set.seed=1234`.

Table 5

For hypotheses $H_1 : \mu_1 > \mu_2 > \mu_3$ vs H_c, the required sample size N per group, and the corresponding $\eta_1 = P(\text{BF}_{1c} > 3|H_1)$ and $\eta_c = P(\text{BF}_{c1} > 3|H_c)$.

effect size		$f = 0.1$				$f = 0.25$				$f = 0.4$			
η		0.80		0.90		0.80		0.90		0.80		0.90	
type of ANOVA	hypotheses	N	η_1/η_c	N	η_1/η_c	N	η_1/η_c	N	η_1/η_c	N	η_1/η_c	N	η_1/η_c
equal	H_1	174	0.801	274	0.901	28	0.805	45	0.904	12	0.821	18	0.910
	H_c		0.902		0.965		0.902		0.968		0.919		0.970
unequal	H_1	179	0.803	283	0.901	29	0.906	46	0.903	12	0.819	18	0.904
	H_c		0.856		0.937		0.859		0.939		0.869		0.940
robust	H_1	203	0.802	323	0.903	33	0.803	51	0.901	13	0.806	20	0.900
	H_c		0.850		0.938		0.845		0.935		0.829		0.935

Note: in this table, the fraction $b = \frac{1}{K}\frac{J}{N}$ is used because the results are independent of the choice of b (Mulder, 2014).

Table 6

For hypotheses $H_0 : \mu_1 = \mu_2 = \mu_3$ vs $H_1 : \mu_1 > \mu_2 > \mu_3$, when the within-group variances are unequal, the distribution of data is non-normal, and $\eta = 0.8$, the required sample size N per group, and the corresponding $\eta_{0,ROB} = P(\mathrm{BF}_{01,ROB} > 3|H_0)$ and $\eta_{1,ROB} = P(\mathrm{BF}_{10,ROB} > 3|H_1)$.

effect size			$f = 0.1$		$f = 0.25$		$f = 0.4$	
results			N	$\eta_{0,ROB}/\eta_{1,ROB}$	N	$\eta_{0,ROB}/\eta_{1,ROB}$	N	$\eta_{0,ROB}/\eta_{1,ROB}$
$b = \frac{1}{K}\frac{J}{N}$	$\gamma = 0.61, \kappa = 0.67$	H_0	735	0.997	90	0.976	30	0.924
		H_1		0.802		0.806		0.828
	$\gamma = 1.75, \kappa = 5.89$	H_0	719	0.996	95	0.974	30	0.922
		H_1		0.801		0.820		0.816
	$\gamma = 0, \kappa = 6.94$	H_0	863	0.998	105	0.982	35	0.940
		H_1		0.801		0.818		0.836
$b = \frac{1}{K}\frac{2J}{N}$	$\gamma = 0.61, \kappa = 0.67$	H_0	674	0.994	80	0.951	25	0.850
		H_1		0.800		0.823		0.838
	$\gamma = 1.75, \kappa = 5.89$	H_0	646	0.988	80	0.947	25	0.847
		H_1		0.801		0.809		0.824
	$\gamma = 0, \kappa = 6.94$	H_0	785	0.996	90	0.957	30	0.874
		H_1		0.804		0.816		0.845
$b = \frac{1}{K}\frac{3J}{N}$	$\gamma = 0.61, \kappa = 0.67$	H_0	625	0.989	70	0.912	26	0.807
		H_1		0.802		0.817		0.871
	$\gamma = 1.75, \kappa = 5.89$	H_0	595	0.982	70	0.905	26	0.803
		H_1		0.801		0.807		0.861
	$\gamma = 0, \kappa = 6.94$	H_0	730	0.994	80	0.932	25	0.804
		H_1		0.804		0.814		0.836
